# Retrieval practice and spaced learning: preventing loss of knowledge in Dutch medical sciences students in an ecologically valid setting

**DOI:** 10.1186/s12909-021-03075-y

**Published:** 2022-01-26

**Authors:** Stijn C. M. Donker, Marc A. T. M. Vorstenbosch, Martin J. T. Gerhardus, Dick H. J. Thijssen

**Affiliations:** 1 Radboud Institute for Health Sciences, Department of Physiology, Radboud University Medical Center, Philips van Leydenlaan 15, 6525 EX Nijmegen, The Netherlands; 2 Radboud Institute for Health Sciences, Department of Anatomy, Radboud University Medical Center, Nijmegen, The Netherlands; 3 Radboud Institute for Health Sciences, Department of Primary and Community Care, Radboud University Medical Center, Nijmegen, The Netherlands

**Keywords:** Knowledge retention, Medical education, Progress testing, Retrieval practice, Spaced learning, The forgetting curve

## Abstract

**Introduction:**

Knowledge, once acquired, degrades over time. Exams that contain questions related to previously acquired knowledge (‘retrieval practice questions’) may promote retrieval practice and spaced learning, and subsequently prevent knowledge loss. To investigate this hypothesis, we compare the score of retrieval practice questions to regular questions in exams of a two-year (bio)medical study program.

**Methods:**

The two-year “Mechanisms of Health and Disease”-program for biomedical sciences and medical students in Nijmegen (the Netherlands) contains 14 spaced exams of 80 questions each. The percentages of correct-, false-, and non-answers were compared between regular questions and retrieval practice questions. Using Pearson correlations between question scores and exam scores (*RiT*-values), the impact of retrieval practice questions on the internal consistency of exams was determined. Mixed model analyses determined changes in outcomes across time.

**Results:**

Analysis of 2006 regular questions and 1728 retrieval practice questions revealed a significantly higher percentage of correct and false answers, and a significantly lower percentage of non-answers, in retrieval practice questions versus regular questions (all *P* < 0.05). Scores did not change across time. *RiT*-values were slightly lower in retrieval practice questions, with a small inverse trend across time.

**Conclusion:**

Our data indicate preservation of knowledge, possibly related to retrieval practice and/or spaced learning. Although the *RiT*-values of retrieval practice questions were slightly lower than those of regular questions, the discriminative capacity was well within acceptable range. These data highlight the potency of retrieval practice questions to prevent knowledge decrement, without altering exam quality.

**Supplementary Information:**

The online version contains supplementary material available at 10.1186/s12909-021-03075-y.

## Introduction

One of the main goals of education is the retention of (newly attained) knowledge. An effective way to achieve knowledge retention is repetitively reviewing study material on multiple occasions [1, 2]. Examination is commonly used to evaluate this learning process. Most educational programs examine students’ knowledge of course material once or twice only. If students do not utilize the acquired knowledge after completion of the course, knowledge is gradually lost. The rate of knowledge loss, also referred to as the ‘forgetting curve’, was first documented by Ebbinghaus in 1913 (Appendix 1) and the presence of this phenomenon has been replicated on numerous occasions, including under various conditions [3].

Examination stimulates students to recall and thus utilize knowledge, where recalling knowledge in itself improves the retrieval of the same material in a later retrieval attempt [1, 4, 5]. Recalling knowledge in the context of preparation for exams is referred to as retrieval practice. Interestingly, retrieval practice leads to superior long-term accessibility of knowledge compared to re-studying (e.g. re-attending a lecture) alone [6, 7], provided that the retrieval attempt was successful [8, 9]. Additional to retrieval practice, it is important to consider the spacing effect as a strategy to optimize retention. A meta-analysis by Cepeda et al. showed that longer intervals between learning (i.e. spacing across weeks or months) potentially cause greater learning effects [10]. In a later study, they demonstrated that the optimal length between two study bouts depends on the time between the last study bout and the final test [2]. Moreover, learning outcomes are superior upon repeated retrieval compared to single retrieval [11, 12]. Although this knowledge suggests that repeated retrieval practice (with sufficient spacing) prevents knowledge loss, little work explored this concept in a real-world setting.

The abovementioned suggests that strategies that promote retrieval practice and spaced learning may be helpful to improve knowledge retention in medical and biomedical sciences students. Recalling knowledge represents an approach that can be actively controlled and influenced by teachers, ultimately leading to improved long-term knowledge retention for students [13]. The importance of medical students’ understanding of basic scientific and medical principles has been documented [14] and attempts to counteract forgetting this knowledge contribute to a more successful curriculum.

Since 2015, the medical faculty of Radboud University (Nijmegen, the Netherlands) implements repeated retrieval practice in the examination of a two-year educational program called “Mechanisms of Health and Disease” (MHD). MHD covers basic scientific and medical principles built from six disciplines: anatomy, psychology, physiology, cell biology, biochemistry and genetics. The program is divided into eight periods, containing one or two exams each. About half of these exams’ questions relate to knowledge obtained in the current period (regular questions (RQs)), whilst the other half relates to knowledge obtained in previous periods (retrieval practice questions (RPQs)). Consecutive exams can contain RPQs related to the same knowledge. Hence, certain knowledge may be examined multiple times (i.e. increased retrieval practice). Anecdotally, annual evaluation cycles indicate that students deliberately re-study course material from previous periods, implying that students adopt a study strategy with increased “spaced” learning. RPQs may have altered students’ learning behavior.

Therefore, the main purpose of our study is to evaluate whether knowledge obtained in the current period (assessed with RQs) alters across subsequent periods when knowledge is recalled through RPQs. Specifically, this retrospective cohort study aims to compare RPQ-scores (across multiple repetitions) to scores of RQs, obtained by medical students and biomedical sciences students, when following the Radboud university medical faculty’s MHD program. Secondly, we aim to determine the effect of RPQs on exam quality by comparing the contribution of RPQs (across multiple repetitions) versus RQs to the internal consistency of exams. This will be done by using the *RiT*-score (indicating how an individual question correlates with the overall exam score) to assess the discriminative capacity of RPQs and RQs. We hypothesize that the RPQs-score does not significantly attenuate across consecutive repetitions, whilst the discriminative capacity of RPQs does not attenuate compared to RQs and across number of repetitions. Accordingly, this work will provide relevant insight into the potential impact of RPQs on assessment quality and its role in the preservation of knowledge.

## Methods

### Study design

In this retrospective cohort study, we analysed exam results of medical students and biomedical sciences students. Data collection commenced in September 2015 and continued until March 2020.

All questions were grouped based on the course they refer to and received the label regular questions (RQs) or retrieval practice questions (RPQs). Whether a question was labelled as RQ or RPQ depended on whether it was the first time a certain topic was examined. For example, from a pool of 50 questions all related to the same topic, three questions were randomly added in consecutive exams. The first time that these questions appeared in an exam, the students attended the course related to that topic the weeks before. These questions were labelled as RQs, meaning that it was the first time that the topic was examined. When the students received questions related to that same topic in subsequent exams, the questions were labelled RPQs. Each question can only be drawn from the question pool once, meaning that each exam is unique and contains different questions.

Additionally, By establishing how many times a certain course has been examined (see “*N of repetitions*” under “outcome measures”), fluctuations in students’ knowledge levels were mapped.

#### Available data

All data used in this study originate from the examination of the 2-year MHD program. The MHD program is obligatory for first- and second-year medical students and biomedical sciences students and over 400 students are enrolled every year. The program is split into 8 periods (period 1 – period 8, P1-P8) of 10 weeks. Exams involve both RQs and RPQs and take place twice in every period with exception of P7 and P8, where examination occurs once and involves solely RPQs. The examined knowledge is cumulative, meaning that questions related to newly taught courses enter the exams as the program continues, while questions related to course material from past courses keep re-appearing. All exams used in the present study are original exams administered between September 2016 and March 2020**.** Resits were not included to prevent selection bias, as resits are made by a non-representative selection of students who already participated in the original exam. Since data are available from September 2016 until March 2020, we were able to include a total of 4080 unique multiple-choice questions (RPQs *n* = 1950, RQs *n* = 2130) from 51 exams (80 questions per exam) (Table 1). Within two weeks after each exam, evaluation took place based on comments from students and scores of individual questions, whereupon formula scoring (i.e. correct minus incorrect answers) was used to grade the exams (for more background: [15]). When ill-formulated or otherwise incorrect, questions were removed from the final calculation of the exam results. The present study did not take these questions into consideration. Microsoft Excel 2016 (Microsoft Corporation, Seattle, WA, USA) was used to compile the database.

**Table 1:** Overview available data

*Period*	*P1*		*P2*		*P3*		*P4*		*P5*		*P6*		*P7*	*P8*
*Exam*	*1*	*2*	*1*	*2*	*1*	*2*	*1*	*2*	*1*	*2*	*1*	*2*	*1*	*1*
*2016–2017* *(% of RPQ’s)*	25	25	62.5	25	62.5	25	62.5	25	62.5	25	62.5	25	100	100
*2017–2018* *(% of RPQ’s)*	25	25	62.5	25	62.5	25	62.5	25	62.5	25	62.5	25	100	100
*2018–2019* *(% of RPQ’s)*	25	25	62.5	25	62.5	25	62.5	25	62.5	25	62.5	25	100	100
*2019–2020* *(% of RPQ’s)*	25	25	62.5	25	62.5	NI	NI	NI	62.5	25	62.5	25	NI	NI

*Note:* Every exam consists of 80 questions. In every academic year, both first- and second-year students are examined. Hence, all exams of the 2-year program are administered every single academic year

NI: Data not included (the present study commenced in March 2020; exams of later date were not included). RPQ’s: retrieval practice questions

#### Outcome measures

All parameters below represent information related to individual questions. The outcomes originate from the standard item analysis and are available for each question included in our database.

#### Percentage of correct question responses (%Cor)

*%Cor* indicates the percentage of correct question responses and is calculated by 100 * [*N* of correct answers] / [*N* of all participating students]. This parameter was used as the indicator of students’ knowledge level. Over-time, changes in *%Cor* reflect changes in the amount of retrievable knowledge (i.e. knowledge preservation).

#### Percentage of non-answers (%Open) & percentage of false question responses (%False)

Within the calculation of the score for the exam, formula scoring is adopted. This procedure is designed to reduce multiple-choice test score irregularities due to guessing. A formula score is obtained by subtracting a predefined score for each incorrect answer, which equals 1 / ([*N* of answer options] – 1). Consequently, students may prefer to not answer a question. The percentage of non-answers (*%Open*) and the percentage of false answer (*%False*) are reported separately. Their calculation is congruent to *%Cor*, with *%Open* = 100 * [*N* of non-answers] / [*N* of all participating students] and *%False* = 100 * [*N* of false answers] / [*N* of all participating students].

## Item total correlation (*RiT*)

Exam questions have to be of reasonable (thus comparable) difficulty and need to discriminate between students with different knowledge levels. The item total correlation (*RiT*) of a question refers to the calculation of the Pearson correlation between the scores for a specific question versus the total scores of the exam, and indicates the grade of adherence to the quality requirements mentioned above. In other words, a high (positive) *RiT* indicates that a high score on the individual question is strongly and positively associated with a high overall exam-score, but also that a low score on the individual question is associated with a low overall exam-score. This means that the individual question is able to successfully discriminate between students with a low versus a high exam score. A low (or negative) *RiT* shows poor discriminative capacity, which may be caused by questions being ambiguous, too simple, or too difficult. We used *RiT* to analyze the effect of RPQs on the internal consistency of the exams and to conclude whether RPQs are of similar quality as RQs.

### Respondents

This parameter indicates the number of individuals that submitted any answer to the question (=[*N* of correct answers] + [*N* of false answers] + [*N* of non-answers]). It represents the size of the response-pool.

### N of answer options

This parameter describes the distribution different numbers of answer options for questions. The questions investigated have two, three, four or five answer options. The outcome of this variable is a count of the different cases.

### Origin of question

The origin of a question indicates to which period the question relates. For example, if *Origin of question* = 1, the question relates to knowledge that was part of the course in P1.

### Period of exam

**Fig. 1:**
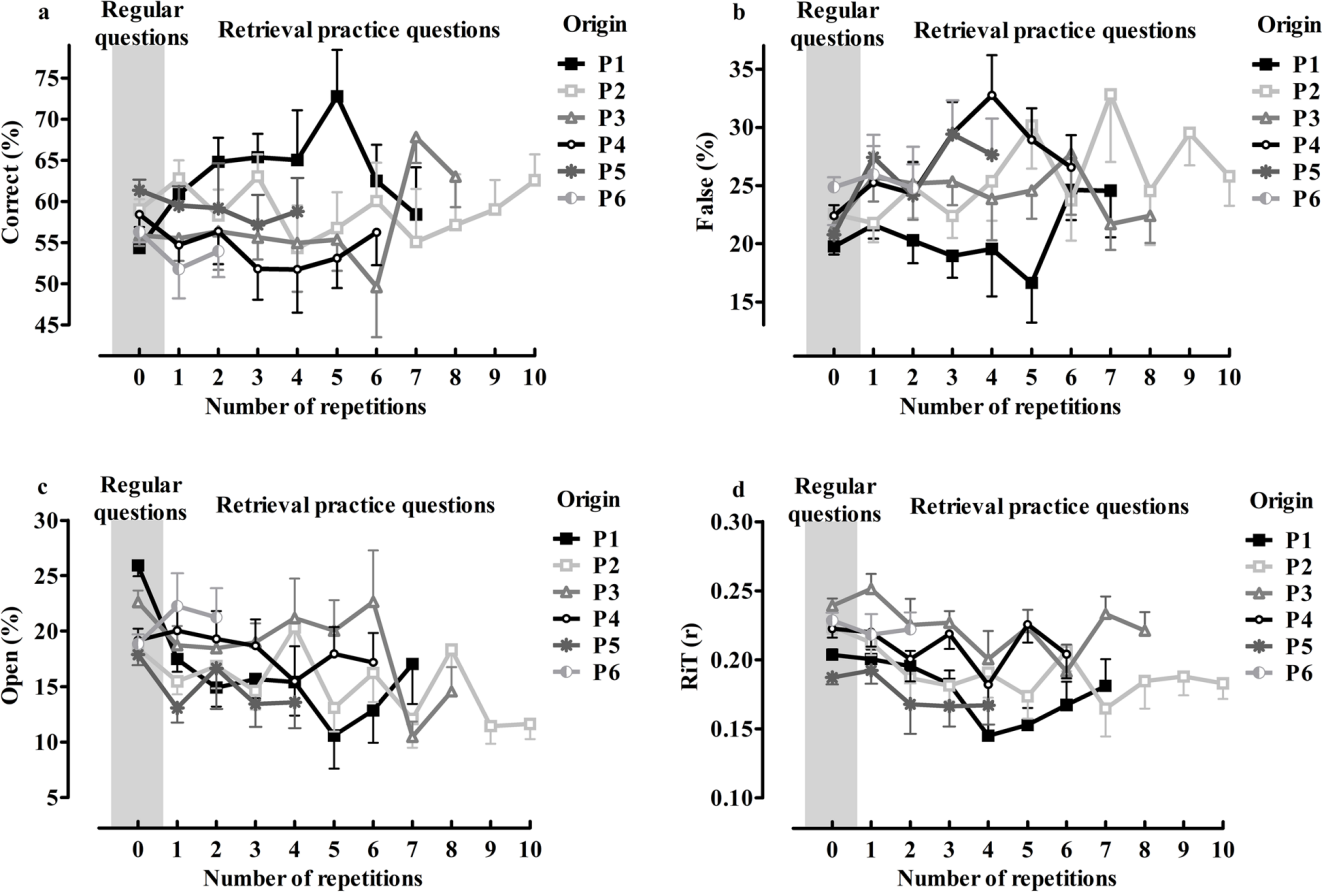
*%Cor* (graph A), *%False* (Graph B), *%Open* (graph C) and *RiT* (graph D) are plotted against N of repetitions. Questions are grouped (separate lines) based on their origin within the ‘mechanism of health and disease’-program related to the six distinct periods in which new knowledge is introduced to students. The first data point of a line (repetition zero) represents the RQs, whilst consecutive data points of the same line (repetition 1–10) represent the RPQs. Mixed model analyses were used to analyse trends of changes in *%Cor*, *%False*, *%Open* and *RiT* in relation to the number of repetitions. Error bars represent standard error of the mean and are plotted on one side of the data points for aesthetical reasons

This variable indicates in which period a question was administered (as part of an exam). Each period contains exam questions with a variety of origins and thus with different *N of repetitions* (e.g. exams in P2 contain questions related to P2, *N of repetitions* = 0; and P1, *N of repetitions* = 1)). *Period of exam* is therefore not a suitable predictor to explore knowledge preservation. It does however represent trends of different periods’ questions and the longitudinal character of this study when questions are grouped based on origin (Fig. 1).

### N of repetitions

The *Origin of question* as well as *Period of exam* of each question is known. It can therefore be calculated what number of retrievals the specific question represents. If a question belongs to the second exam of a period, *N of repetitions* was calculated by (*Period of exam* – *Origin of question*) * 2. If the question belongs to the first exam of a period, 1 was subtracted from the outcome of that formula. For example, the *N of repetitions* of P2 questions in the first exam in P5 = ((5–2)*2)-1 = 5. This means that the material from P2 is examined for the fifth time in exam 1 of P5. If the exam occurred in P8, 2 was subtracted from the formula since P7 only contains 1 exam. Possible values are all integers between and including zero to ten, with zero being all RQs.

#### Data analysis

Analysis primarily focused on exploring the relationship between *N of repetitions (0 = RQs, 1–10 = RPQs)* on question score and question quality. Accordingly, *%Cor* and *RiT* were our main response variables. *N of repetitions* as a predictor essentially causes normalization of the data: it causes data to overlap as all questions (regardless of their origin) are introduced for the first time at x = 0 (zero repetitions = RQs). To assess question score over time, the relationship between *N of repetitions* and *%Cor* was determined using linear mixed model analysis. By using the origin (to which period a question relates) of a question as a grouping variable, *%Cor* across *N of repetitions* was assessed for each origin individually before estimating the size of the fixed effect (*N of repetitions*). We analyzed the relationship between *N of repetitions* and *%False* and *%Open* in a similar way to the analysis of *%Cor*, to provide full disclosure of the distribution of question feedback. To determine the effect of RPQs on internal consistency of the exams, the *RiT* of RQs versus RPQs was compared using a Mann-Whitney U test. To assess a possible relation with *N of repetitions* we also performed a mixed model analysis using *RiT* as a dependent variable.

A descriptive section in which the pool of RPQs is compared with the pool of RQs is included in this paper (Table 2). The distribution of *N of answer options* was statistically analyzed using a Chi-Square test. Significant Shapiro-Wilk and Kolmogorov-Smirnoff tests indicated that none of the other variables were normally distributed (a common finding in psychometrics). Hence, these variables were analyzed using non-parametric Mann-Whitney U tests.

**Table 2:** Question characteristics

*Question category*		RQs *(N* = 2006)	RPQs (*N* = 1728)	*P*-value	
*N* of answer options	2	261	228	*P* = 0.740	
	3	1059	926		
	4	663	549		
	5	23	25		
Respondents (*M* ± *SD*)		430 ± 21	415 ± 36	*P* < 0.001	
%Cor (*M* ± *SD*)		57 ± 24	59 ± 24	*P =* 0.035	
%False (*M* ± *SD*)		22 ± 15	25 ± 17	*P* < 0.001	
%Open (*M* ± *SD*)		21 ± 18	16 ± 15	*P* < 0.001	
RiT (*M* ± *SD*)		0.22 ± 0.10	0.20 ± 0.09	*P* < 0.001	

*Note:* Values represent frequency of questions unless indicated otherwise*.* RPQs: questions related to formerly studied matter; RQs: regular questions; RiT: Item total correlation

Statistics were performed using Statistical Package for the Social Sciences (SPSS) 26 (International Business Machines Corporation, Armonk, NY, USA) and GraphPad Prism 5.03 (GraphPad Software Inc., La Jolla, CA, USA).

## Results

A total of 196 questions were excluded from the database as they were not included in the final grade calculation, and 150 questions were excluded as they related to high school knowledge. The remaining 3734 questions were used for analysis (RPQs *n* = 1728, RQs *n* = 2006).

### RQs versus RPQs

In Table 2, questions are divided into two categories: RQs and RPQs. The distribution of numbers of answer options between the groups was not significantly different. On average, RPQs had fewer respondents than RQs (*P* < 0.001). *%Cor* and *%False* were significantly higher in RPQs compared to RQs (*P* < 0.05 and *P* < 0.001, respectively). RPQs had a significantly lower *%Open* and *RiT* (both *P* < 0.001) than RQs.

### Outcomes as function of N of repetitions

Figure 1 illustrates *%Cor* (graph A), *%False* (graph B) and *%Open* (graph C) for separate origins across all periods of the MHD-program. Linear mixed model revealed no significant change in %Cor across the number of repetitions (β = .186, *P* = .73). The analysis also showed no significant association between number of repetitions and *%False* (β = .589, *P* = .07), or *%Open* (β = −.933, *P* = .08). Graph D in Fig. 1 depicts *RiT* across number of repetitions. A minor, but significant trend was found in *RiT* across number of repetitions (β = −.004, *P* < 0.001). In Appendix 2, the same outcome measures are depicted in a table, but irrespective of the origin of questions.

## Discussion

Our retrospective study compared the scores of regular questions (RQs) to retrieval practice questions (RPQs) across a two-year program on ‘mechanisms of health and disease’ for medical students and students in biomedical sciences. It explored the relation of these questions with the overall exam score. This work presents the following findings. First, compared to RQs, the RPQs received a small but significantly higher proportion of correct *and* false answers, and subsequently a significantly lower number of questions that were left open. This indicates that students were more likely to answer RPQs, with preservation of the mean score for the questions. Second, time-dependent analysis of RPQs revealed no significant change across multiple repetitions, supporting the absence of the ‘forgetting curve’. This suggests preservation of knowledge within this investigated program. Third, the discriminative capacity of RPQs, quantified as the *RiT*, was slightly but significantly lower than RQs. Fourth, time-dependent analysis revealed a small, inverse relation between the number of repetitions and *RiT*-score across multiple repetitions (*n* = 10). Despite the significantly lower discriminative capacity of RPQs, the magnitude of this decline remained within the limits of acceptable internal consistency of the exam. Altogether, our data indicate that the use of RPQs across a two-year educational program prevents significant loss of knowledge during that program, without affecting exam quality.

Comparing the outcomes for RPQs and RQs, we found no evidence for a decline in scores when repeatedly testing knowledge. In accordance with other research (e.g. Custers & ten Cate, 2011), we linked the percentage of correct answers to knowledge levels and knowledge retention. Importantly, the exam set-up warrants caution in interpreting the percentage of correct answers, because students were allowed to leave questions open. This option prevents ‘correction for guessing’, as an incorrect answer leads to subtracting points from the exam score. We found that both the percentage of correct *and* false answers are increased in RPQs, which logically coincides with a decline in the percentage of non-answer (*%Open*). Consequently, the small increase in *%Cor* does not simply imply improved knowledge retention. A gain in confidence of respondents could be present as they proceed in the program, resulting in a slightly higher percentage of correct, but also incorrect answers for RPQs. At the very least, the lack of a significant decline in score for the RPQs across multiple repetitions indicates no knowledge loss over time, and a successful prevention of the characteristic ‘forgetting curve’ for the duration of the MHD program.

A notable loss of knowledge occurs after its acquisition. Virtually all information, including daily life situations, is subject to the characteristic forgetting curve (e.g. eye colour of your best friend, last nights’ dinner), often within days to weeks [3, 16, 17]. The forgetting curve is also applicable, albeit within months to years, to the learning material of (bio)medical study programs [13, 18]. Methods like retrieval practice and spaced learning may limit this forgetting [6, 7, 10]. The MHD program facilitates retrieval practice through RPQs and leads to a more spaced learning approach, which likely contributes to the absence of knowledge loss within the investigated MHD program.

Furthermore, we suggest that our results can be explained using the dual-memory theory [19]. This theory states that studying alone strengthens an *existing* memory trace, whereas testing leads to the formation of a *new* memory trace [20]. During the repeated examination of students in the MHD program, these new memory traces could have been formed. Thus, the students’ memory of studying the material could have been complemented with a second memory of that material being examined, which would have logically benefited knowledge levels and consequently question scores.

An alternative explanation for the preservation of knowledge is that the acquired MHD knowledge is repeated or is built upon in subsequent periods within the MHD-program and/or within the broader spectrum of the curriculum. The structure of the biomedical curriculum as a whole may therefore prevent knowledge decrement, as additional learning reactivates related memories [21]. Interestingly, the last 2 periods of the MHD-program (i.e. P7 and P8) do not introduce new knowledge, but promote the utilization of previously acquired MHD-related knowledge. Except for RPQs originating from P3 (Appendix 3), there is no improvement in exam scores during P7 or P8 (Fig. 1). Therefore, the structure of the curriculum unlikely represents the only explanation for the preservation of knowledge, as one may expect also the score for RQs to increase upon subsequent periods. Altogether we conclude that, although the exact cause of the preservation of knowledge cannot be identified, the absence of significant changes suggests that knowledge retention may be explained by retrieval practice and spaced learning, characteristic for the MHD-program.

Our second aim was to investigate whether the discriminative capacity of the RPQs differed from RQs. In contrast to our hypothesis, we found a significantly lower *RiT*, reflecting a lower contribution of RPQs to the internal consistency of an exam. Furthermore, the marginal but significant decline across number of repetitions suggests that the discriminative capacity of RPQs declines with more repetitions. A possible explanation could be an increase in confidence regarding repeatedly examined course material in all students. Therefore, responses to RPQs may be related to students’ overall exam scores to a lesser extent than before, causing a slight decrease of *RiT* in RPQs. Although these observations imply that RPQs negatively impact the capacity of exams to discriminate between ‘good’ and ‘bad’ students, caution is warranted. Previous work recommends to aim for *RiT*-values of at least .15 [22]. Since our RQs have a *RiT* of 0.22, only 0.02 higher than RPQs, we question the relevance of this small difference for the internal consistency of the exam. This is further supported by the relatively small variation in *RiT*-values for both types of questions. Indeed, the 95% confidence interval for *RiT*-values of RPQs was .197 to .206 versus .212 to .220 for RQs, which is all well above the mentioned .15 minimum, being classified as non-harmful to exams’ internal consistency. The minor difference in *RiT* between RPQs and RQs means that students do not pass exams based on their knowledge of past periods only and need to obtain sufficient knowledge related to both previous and current period(s).

### Limitations

An important limitation to highlight for this study is the structure of the exam, where students had the possibility to not answer questions. This complicated the interpretation of our data, especially the question whether and how leaving questions open relates to knowledge preservation (or loss). Nonetheless, the relatively small change in questions that were left open allowed us to robustly examine our primary research question. Another obvious limitation is the lack of a control group to truly examine the role of the RPQs. This is important since, in contrast with most studies [3, 13, 16, 18], some found little knowledge decrement during the first 2 years after medical knowledge has last been studied [23]. However, the latter study adopted a cross-sectional design and, more importantly, individuals reported to be exposed to retrieval practice.

### Conclusion

In conclusion, although one of the main goals of education is the retention of knowledge, teaching and exams are often followed by loss of knowledge (i.e. the forgetting curve). Our study examined the proportion of correct answers to questions within the cumulative examination strategy of the ‘mechanisms of health and disease’-program for medical and biomedical sciences students. In line with the hypothesis that retrieval practice questions promote retrieval practice and a more spaced learning approach, we found no decrement in scores for retrieval practice questions across the two-year educational program. It implies that the cumulative examination strategy may be an effective strategy in counteracting the forgetting curve. The cumulative aspect of the examination through the RPQs did not importantly decrease overall quality of the exams. This work highlights the potential of a cumulative examination approach to promote knowledge retention in medical and biomedical sciences students, by utilizing the principles of retrieval practice and spaced learning.

## Supplementary Information

**Additional file 1.**

## Abbreviations

*%Cor*: Percentage of correct answers (100 * [*N* of correct answers] [*N* of all participating students])
*%Open*: Percentage of non-answers (100 * [*N* of non-answers] / [*N* of all participating students])
*%False*: Percentage of false answers (100 * [*N* of false answers] / [*N* of all participating students])
MHD: Mechanisms of health and disease
P1-P8: Period 1–8
*RiT*: Item total correlation; correlation between item score and total exam score.
RPQs: Retrieval practice questions; questions related to previously taught courses
RQs: Regular questions; questions related to the current course.
