# Supplementary material for: Retrieval practice and spaced learning: preventing loss of knowledge in Dutch medical sciences students in an ecologically valid setting
Source: BMC Med Educ. 2022 Jan 26;22:65. doi: 10.1186/s12909-021-03075-y (PMC8793259; doi:10.1186/s12909-021-03075-y)
Supplement: Supplementary file 1 — Additional file 1. [file 12909_2021_3075_MOESM1_ESM.docx]

# Appendix 1


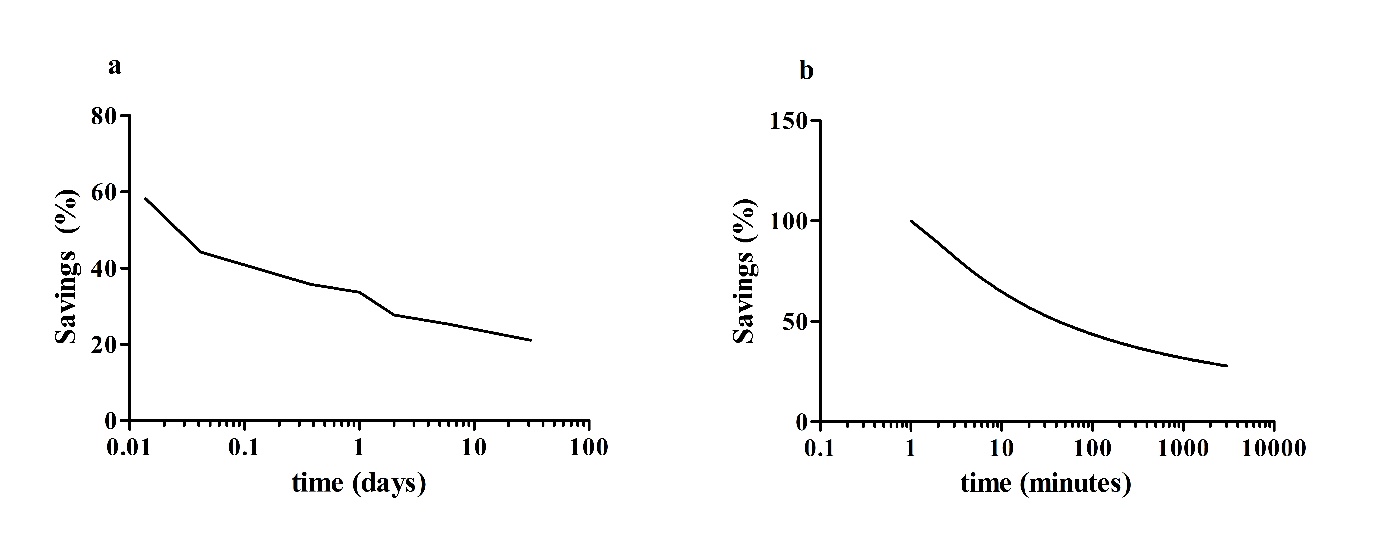
The results of Ebbinghaus' experiment (a) and the famous forgetting curve using his formula (*b*=100*k*/((log *t*)*^c^*+k) with *k*=1.84 and *c*=1.25) (b) [3]. The percentage of saved knowledge (Y-axis) is plotted against time on a logarithmic scale (X-axis).

# Appendix 2

| *N of repetitions* | *0 (=RQs)* (*N*=2006) | *1*  (*N*=578) | *2*  (*N*=257) | *3*  (*N*=301) | *4*  *(N*=113) | *5*  *(N*=135) | *6*  (*N*=102) | *7*  (*N*=83) | *8*  (*N*=65) | *9*  (*N*=46) | *10*  (*N*=48) |  |  |  |  |  |
| --- | --- | --- | --- | --- | --- | --- | --- | --- | --- | --- | --- | --- | --- | --- | --- | --- |
| Origin of questions | P1-P6 | P1-P6 | P1-P6 | P1-P5 | P1-P5 | P1-P4 | P1-P4 | P1-P3 | P2, P3 | P2 | P2 |  |  |  |  |  |
| %Cor (*M*) | 57 | 59 | 59 | 59 | 57 | 57 | 57 | 63 | 61 | 59 | 63 |  |  |  |  |  |
| %False (*M*) | 22 | 24 | 23 | 24 | 27 | 27 | 26 | 25 | 23 | 30 | 26 |  |  |  |  |  |
| %Open (*M*) | 21 | 17 | 18 | 16 | 16 | 16 | 17 | 12 | 16 | 11 | 12 |  |  |  |  |  |
| RiT (*r*) | .22 | .21 | .20 | .20 | .18 | .20 | .20 | .21 | .21 | .19 | .18 |  |  |  |  |  |
| *Note:* Questions are grouped based on *N of repetitions*. All RQs are questions related to new course material, hence *N of repetitions*=0 for RQs. *Origin of questions* indicates which questions are repeated *N* times (e.g. questions originating from P2 are repeated 10 times). P: period. | | | | | | | | | | | | | | | | |

Number of repetitions, characteristics

#
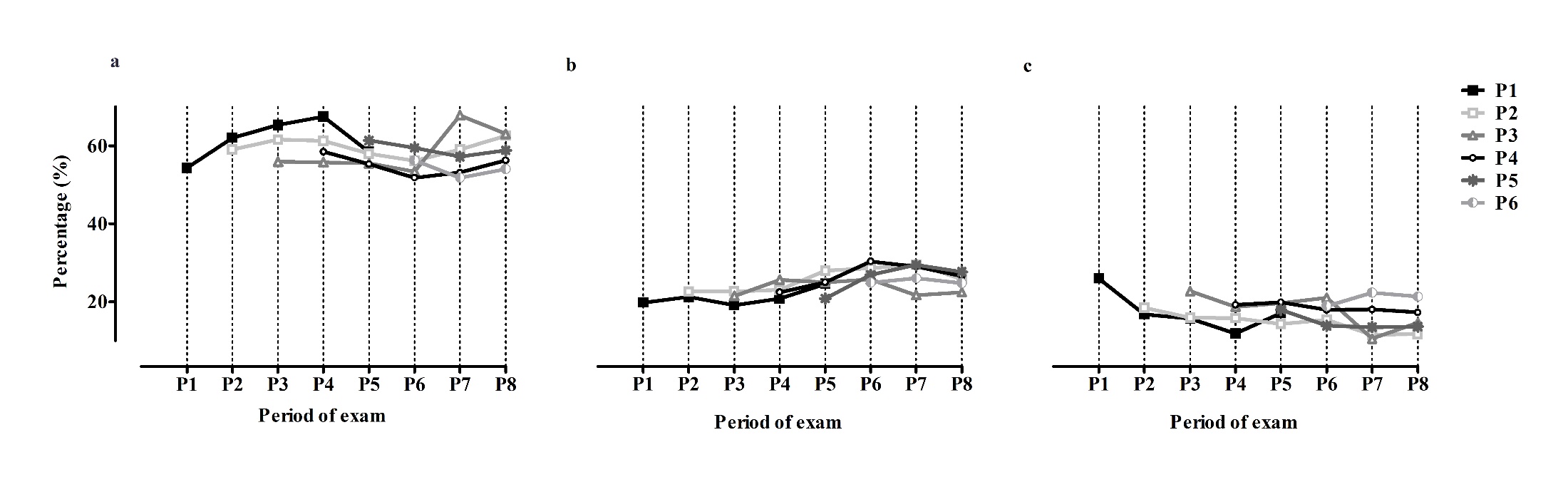
Appendix 3

%Cor (graph A), %False (graph B) and %Open (Graph C) are plotted against the period of examination. Exams take place twice in P1-P6 and once in P7 and P8. Questions are grouped (separate lines) based on their origin. The lines represent the introduction of questions related to one topic and their slope implies changes in scores across consecutive periods (=time). E.g. in P2, questions related to P2’s course material are introduced for the first time and keep re-appearing in exams until P8. The first datapoint of a line represents RQs, consecutive datapoints of the same line represent RPQs. This figure represents the longitudinal character of the MHD program, but is not suitable to analyse knowledge preservation across the complete data set as each group of questions has a different onset in the curriculum.
